# Sensor Data Acquisition and Processing Parameters for Human Activity Classification

**DOI:** 10.3390/s140304239

**Published:** 2014-03-04

**Authors:** Sebastian D. Bersch, Djamel Azzi, Rinat Khusainov, Ifeyinwa E. Achumba, Jana Ries

**Affiliations:** 1 School of Engineering, University of Portsmouth, Anglesea Building, Anglesea Road, Portsmouth PO1 3DJ, UK; E-Mails: Djamel.Azzi@port.ac.uk (D.A.); Rinat.Khusainov@port.ac.uk (R.K.); ifeyinwaeucharia@gmail.com (I.E.A.); 2 Portsmouth Business School, University of Portsmouth, Richmond Building, Portland Street, Portsmouth PO1 3DE, UK; E-Mail: Jana.Ries@port.ac.uk

**Keywords:** Ambient Assisted Living (AAL), data acquisition, data sampling, event classification, optimization

## Abstract

It is known that parameter selection for data sampling frequency and segmentation techniques (including different methods and window sizes) has an impact on the classification accuracy. For Ambient Assisted Living (AAL), no clear information to select these parameters exists, hence a wide variety and inconsistency across today's literature is observed. This paper presents the empirical investigation of different data sampling rates, segmentation techniques and segmentation window sizes and their effect on the accuracy of Activity of Daily Living (ADL) event classification and computational load for two different accelerometer sensor datasets. The study is conducted using an ANalysis Of VAriance (ANOVA) based on 32 different window sizes, three different segmentation algorithm (with and without overlap, totaling in six different parameters) and six sampling frequencies for nine common classification algorithms. The classification accuracy is based on a feature vector consisting of Root Mean Square (RMS), Mean, Signal Magnitude Area (SMA), Signal Vector Magnitude (here SMV), Energy, Entropy, FFTPeak, Standard Deviation (STD). The results are presented alongside recommendations for the parameter selection on the basis of the best performing parameter combinations that are identified by means of the corresponding Pareto curve.

## 1. Introduction

**Figure 1.**
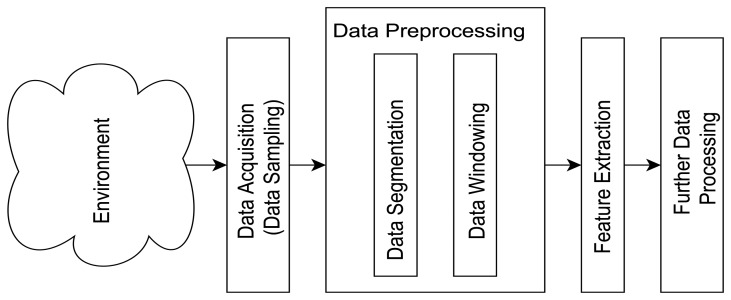
Pre-steps before ADL.

Ambient Assisted Living (AAL) is currently on the research agenda of many stakeholders worldwide, especially in Western countries, driven mainly by the needs of an aging population and in an attempt to address the demands of care and intervention for the elderly and those who require care. The main areas of interest in Assisted Living (AL) include fall prevention, promotion of independence, as well as ambulation and Activity of Daily Living (ADL) monitoring (for fall detection, activity recognition and classification). The timeliness and accuracy of the classification of ADL activities could have severe consequences if inadequate, especially in the case of an emergency event such as a fall and are therefore essential to provide the elderly with a sense of security and confidence [1,2]. Furthermore, reasonable levels of ADL facilitate the promotion of independence, hence the need for ambulation and ADL monitoring. Consequently, automated monitoring of subjects living independently in their homes, using wearable and off-body sensor-based devices, has been the subject of numerous research studies. While the literature highlights a great number of research areas for assisted living, such as sensor designs, placement of monitoring devices, novel monitoring techniques, fall detection and ADL data collection and classification methods, it fails to clarify some of the underlying and fundamental aspects of data collection in this field such as data acquisition and pre-processing (outlined in Figure 1, presenting standard prerequisites before ADL classification can take place).

Falls and ADL events are generally classified based on the features extracted from segments of the monitoring sensor data and have therefore a significant role in the accuracy of event classification [3]. Even though researchers are aware of the importance of sampling frequency; segmentation method; and window size with respect to feature extraction, the issue is not addressed in the reviewed studies with no clear explanation or justification given for the parameter selection. Furthermore, researchers tend to ignore the required Computational Load (CL) for data classification, which is of particular interest once data classification takes place on an embedded system for real time ADL recognition.

The literature review showed that there is no consensus in the selection of parameter combinations which once chosen, are seldom varied by researchers to improve classification results. Therefore, the work described in this paper empirically investigates the influence of sampling frequency (SF), segmentation method (SM), and windows size (WS) on the classification accuracy (CA) and computational load (CL) using two independent datasets (from Bao *et al.* and Roggen *et al.*). The work presented here tests eight commonly used features that are obtained from the accelerometer sensor data to determine CA and CL. The input information for the classifier are Root Mean Square (RMS), Mean, Signal Magnitude Area (SMA), Signal Vector Magnitude (here SMV), Energy, Entropy, FFTPeak, and Standard Deviation (STD). The results have been analysed using an ANalysis Of VAriances (ANOVA) to reveal the influence of the parameter combinations on the CA and CL. This is followed by an approach to recommend the parameter combinations that achieve the best CA disregarding CL and vice versa. Other parameter combinations may represent interesting trade-off points between these two preferences. This may be required in situations where time and hardware resources are limited. The authors aim to provide a more informed approach to parameter selection for event classification (with respect to the investigated ADLs) in the area of AAL.

Section 2 will highlight existing literature to outline the inconsistency and insufficient justification for parameter selection in ADL classification. This section also presents the process of data acquisition and introduces different segmentation techniques. Section 3 describes the investigation procedure. Section 4 presents the experimental results with a recommendation for parameter combinations, and Sections 5 and 6 present the discussion of results and conclusion.

## 2. Divergence in the Parameter Selection

### 2.1. Sampling Rate

The acquisition of data is one of the most critical steps in event classification as re-running experiments with test subjects is not always possible. Undersampling leads to loss of information and oversampling can result in information buried in unwanted noise. In the latter case, longer computational time is needed for analysis as more data needs to be processed. The minimum sampling rate *f_sampling_* is dependent on the maximum frequency contained in the data signal *f_max_* (the sampling theorem) [4]. In the area of AAL, a review of the literature has not uncovered a typical sampling frequency.

The highest sampling rate for AAL that the authors found during their research is 512 Hz by [5] followed by the works of [6] where the authors use a sampling rate of 256 Hz to collect accelerometer data. [7] use a two-axis accelerometer and a sampling frequency of 76.25 Hz, which is less than 1/3 of [6] sampling rate. [8] choose *f_samplin_*_g_ to be 64 Hz. The authors acknowledge the high frequency sampling rate used by [6] however they reduced the sampling frequency on the bases that lower values are more feasible with off-the-shelf activity monitors. They further mention the work of [9], who sample accelerometer data at 50 Hz, therefore resampling their own data at the same frequency as well. Overall the literature highlights that values around 50 Hz are one of the more common sampling rates. [10] use 52 Hz, [11] use 50 Hz to sample their tri-axial accelerometer, while [12] and [13] also report a 50 Hz sampling rate for an eWatch with two-axis accelerometer and a light sensor. To the authors' best knowledge, [13] are the only ones that tested different sampling frequencies (from 1 to 30 Hz) for the sensor data. The outcome highlights that the recognition of ADLs improves with higher sampling rates but only marginally improves with sampling rates above 20 Hz. In [14] the authors demonstrate that 98% of the FFT spectrum amplitude is contained below 10 Hz, and 99% below 15 Hz. This corresponds to the findings of [15] who state that a sampling frequency of 20 Hz is sufficient to successfully classify ADLs. The lowest sampling rate that the authors found in the literature is 5 Hz by [16].

### 2.2. Data Preprocessing Techniques

#### 2.2.1. Segmentation Method

One of the challenges of data pre-processing following acquisition consists in deciding which points to actually use in the live stream of data. Several different segmentation methods exist to divide a larger data stream into smaller fit for processing chunks. The selection of the right segmentation technique is crucial, as it immediately impacts on the extracted features used for the ADL classification and the resulting classification accuracy. Therefore even the best classifier performance will be weak when the extracted features are non-differentiable [3]. Furthermore, the segmentation techniques can also have an impact on the real time capabilities as complex segmentation methods can increase CL but might result in improved classification accuracy. Moreover, the segmentation method also dictates how often features need to be extracted and classification algorithms need to be executed. Literature has highlighted several different segmentation techniques used in various research projects, such as: Fixed-size Non-overlapping Sliding Window (FNSW) [3,17], Fixed-size Overlapping Sliding Window (FOSW) [3,17], Top-Down (ToD) [17], Bottom-Up (BUp) [17], Sliding Window And Bottom-up (SWAB) [17], Symbolic Aggregate approXimation (SAX) [3], String Matching (SM) [3], Reference-based Windowing (RbW) [18], Dynamic Windowing (DWin) [19] and Variable-size Sliding Window (VSW) [20]. The significant difference in these techniques resides in their online and offline capabilities. The meaning of an online technique is that the data can be segmented before the complete data is collected, while offline methods require the entire dataset first. For real time applications, only online techniques are of interest. [17] note that online algorithms can produce very poor approximations of data under certain conditions but have a relatively good performance on noisy data. However, the authors also highlight that the FOSW segmentation algorithm is of particular interest in medical research, e.g., patient monitoring as the algorithm is simple and intuitive for researchers to understand. As part of this paper the algorithms investigated should be fairly simple to understand and online capable (FNSW, FOSW, and SWAB).

#### 2.2.2. Window Size

Researchers who use fixed size window segmentation methods apply inconsistent window sizes. [10] use especially short windows of 1 s. [8] report to use a 2 s window based on their short ADLs in their research and because they achieve only a minimal gain in classification accuracies with features from a 3 s window. Further examples for short windows are [21] and [9], with 2 s and 2.56 s respectively. [13] extract features from a 4 s buffer, [12] use 5 s in their research, and [7] report a window length of 6.7 s. While these researchers are using short window sizes, [20] describe the usage of a 60 s windows in the work of [22] and 74 s in [23]. Furthermore, [20] introduce possible modification to the fixed size window methods. The authors suggest in their work to dynamically adjust the window size based on special events in the sensor data as different ADLs have different time frames. They raise the point that longer window sizes can cover more than one ADL while a small window could split an activity, which both leads to suboptimal information for an activity classifications algorithm.

The work of [16] has a similar point, indicating that to achieve good classification accuracy, different sensor features should be extracted using varying window sizes. These methods lead to complex monitoring systems if several ADLs need to be classified. Each feature window could yield different ADL classification results, which would then require a voting system to predict the correct ADL from the list of possible activities. Section 2 highlighted the divergence in parameter selection in the literature covering ADL event classification. Table 1 represents a summary of the different combinations discovered. The section above pointed out problems that are introduced when the wrong sampling frequency (over/undersampling) is used for the data acquisition. It also showed that researchers in the field are not in agreement over which sampling rate to use. The section also showed the use of various window sizes covering a wide range of values. Most studies base their parameter combinations on past experiments, hardware limitations, or do not state a specific reason. It was also found that possible CA or CL improvements based on different combinations are neither investigated nor mentioned. This inconsistency in parameter selection is the fundament for the study presented here for a more informed decision on parameter selection.

## 3. Investigation Procedure

This work presented here was based on two different datasets from the literature. The first dataset contains two-axis accelerometer data collected by [7]. The data contains 20 different participants (13 males and seven females with a mean age of 21.8 years (±6.59 years SD)), which were recruited at the MIT with the help of posters. The experiment required the test subjects to execute several different ADLs under laboratory conditions without any supervision or guidance. Sensor data was collected simultaneously at five different positions (ankle, thigh, wrist, hip, upper arm), with a sampling frequency of 76.25 Hz. From the five sensor positions, the data of the hip sensor from all twenty different participants were used with the focus on ADLs such as, walking, sitting, walking and carrying an item, standing still, lying down, and climbing stairs. The second dataset is the Opportunity dataset collected as part of a European Funded project by [24]. The dataset is not limited to just body-worn accelerometer data. The complete set includes a total of 10 different sensor types, such as microphone, magnetometer, UWB localization, RFID, *etc.*, totaling a collection of 72 sensors. Data was recorded with 12 test subjects, which are not further specified. Of these 12 subjects only three subjects are labeled and available in the UCI Machine Learning Database. The labeled locomotion activities that were used from this dataset are stand, walk, sit, and lie. As both datasets should be similar to allow for comparable result, the Opportunity dataset was limited to the accelerometer (sampled at 64 Hz) placed at the subject's hip and limited to the *x* and *y* axis. Using Matlab [25], the sensor data was resampled, segmented and the data features extracted, while the Weka software package [26] provided the implementation of the classification algorithms.

### 3.1. Resampling

Section 2 showed that sampling rates vary greatly throughout the literature; it also indicated a high use of sampling rates around 50 Hz even though the work of Maurer *et al.* argues that sampling rates above 20 Hz only marginally improve classification accuracy [13]. Therefore the complete data set was resampled using Matlab at six different sampling rates in the range of 10 to 60 Hz in 10 Hz steps. Intermediate steps were ignored for the benefit of faster experiments, as well as the authors' belief that the omission of intermediate steps would not cause loss of generality of the results. Additionally, sampling rates above 60 Hz were excluded, as the authors concur with [8], who stated that higher sampling rates are harder to achieve with off-the-shelve-components.

### 3.2. Data Segmentation

The work presented here focuses on three online segmentation techniques: FNSW, FOSW (with four different overlap percentages), and SWAB that were introduced in Section 2.2. As described above, the advantages of these algorithms are that they are online capable, therefore can be used while the data collection is in progress and are simple and intuitive so that they are easily understood.

**Figure 2.**
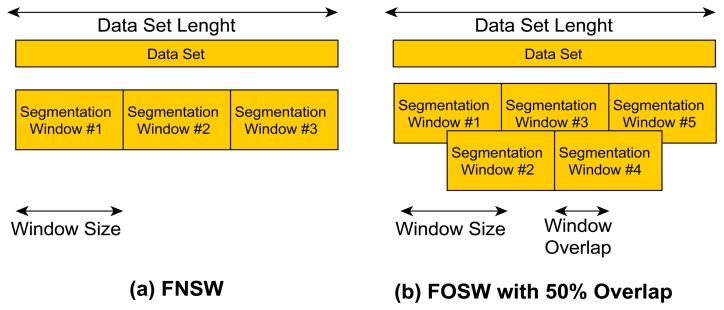
Explanation of segmentation method. (**a**) FNSW; (**b**) FOSW with 50% overlap.

FNSW is a simple segmentation technique without any data overlap (see Figure 2a). The end point of segmentation window *N* is the starting point for window *N* + 1. It is therefore possible to exactly calculate the amount of windows generated for a given data set size with Equation (1):

(1) $${\text{Segmentation}}_{\text{Windows}}=\frac{S}{L_{i}}$$

where S is the total number of signal samples and *L_i_* = R_Sampling_*Window_Size_, where *R_Sampling_* is the data resampling rate used (in the range of 10 to 60 Hz) and *Window_Size_* is the selected window size (in the range of 0.5 to 24 s). One disadvantage of this technique is that data associated with a particular feature (e.g., fall) can be split between windows. A FNSW sliding technique that is not covered in this paper is to leave a gap between adjacent windows, as this would result in uncovered sensor data and therefore could miss important information.

The FOSW segmentation technique is based on FNSW but includes data overlap (see Figure 2b showing FOSW with an overlap of 50%). Depending on the percentage overlap, more or less data overlaps from window *N* into *N* + 1. This is also referred to as a window shift. A 0% overlap corresponds to the FNSW segmentation method, while an overlap of 100% would yield to a static window as it would not be shifted and the data would always be segmented at the exact same point. Therefore, the requirement for FOSW is to move with at least one data point per segmentation. The number of segmentation windows generated can be calculated using Equation (2):

(2) $${\text{Segmentation}}_{\text{Windows}}=\frac{S}{L_{i}-L_{i}*\frac{P_{j}}{100}}$$

With *P_j_* being one of the following percentage overlap values used for this research: 25, 50, 75, and 90.

**Figure 3.**
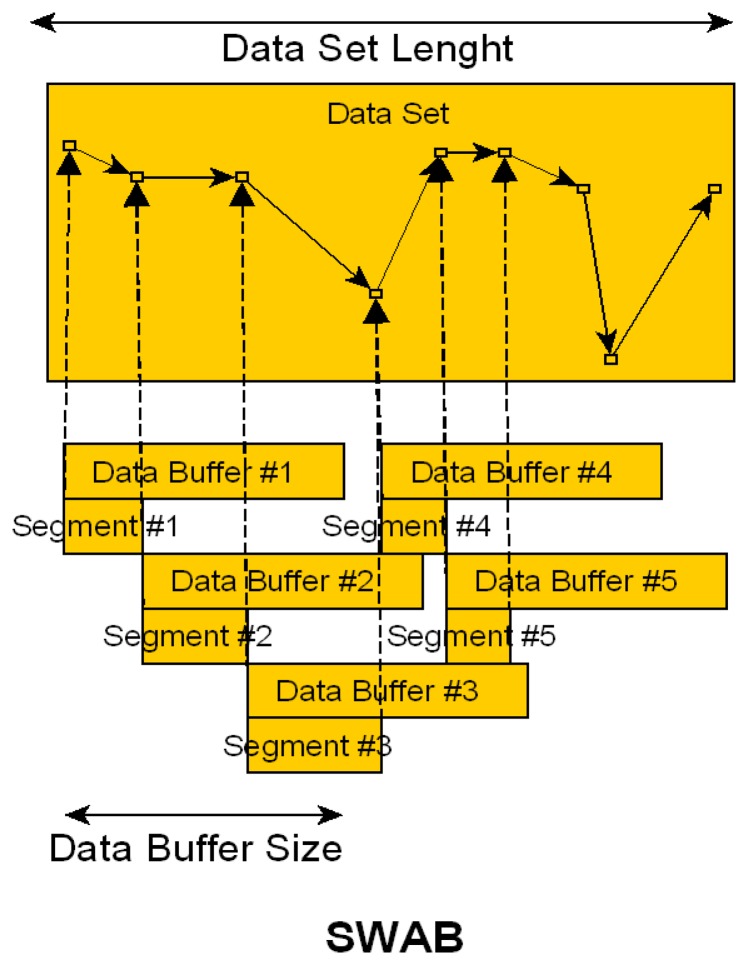
Explanation of segmentation method SWAB.

SWAB is the third segmentation technique used as part of the study presented here and was designed by [17]. It is a combination of the Sliding Window and Bottom-up approach. The process is visualized in Figure 3. The algorithm has a fixed size data buffer that is used for the Bottom Up approximation, which joins the smallest approximation segments until a stopping condition is met. Once the approximation for the window is complete the data buffer shifts by the first segment (here identified as Segment #1 in the illustration) and the process is repeated for the new buffer window. Each segment is used for feature extraction. As the data shift is dependent on the dataset and its approximation, it is not possible to estimate the amount of segmentation windows generated by the algorithm. The implementation is more complex compared to the FNSW and FOSW methods described above and therefore an increased CL is expected while the CA gain is uncertain even though literature suggests improved results.

As highlighted earlier in Section 2.2, there is no clear recommendation in the published literature on the selection of the window size used for the data segmentation. The authors therefore tested a range of 32 different sizes in the range of 0.5 to 24 s. In the area of 0.5 to 8 s, the size is increased in 0.5 s steps, while thereafter the step size is increased to 1 s. The 0.5 s step size was increased after 8 s because the ADLs under investigation have only a short time frame and computational load was reduced for the experiment. Even though literature showed the use of longer window sizes the aim is to only include single ADLs in each window to achieve the best classification results. The authors' initial research [27] supports this idea as it indicates a decrease in accuracy for window sizes above 8 s. Furthermore, it is the authors' belief that most ADLs will only take a short amount of time and a maximum of 24 s should be sufficient to include at least two ADLs.

### 3.3. Data Feature Selection

The following eight metrics are quite common in the area of ADL classification and therefore used to retrieve the different features of the accelerometer sensor data in this research: Root Mean Square (RMS), Mean, Signal Magnitude Area (SMA), Signal Vector Magnitude (here SMV), Energy, Entropy, FFTPeak, Standard Deviation (STD). These metrics and their significance are discussed below as each individual metric has its own influence in the research field.

RMS has been used to distinguish walking patterns [6] as well as being an input to classifiers for activity recognition [16,28]. The RMS value is calculated using Equation (3):

(3) $${\text{RMS}}_{x-\text{Axis}}=\sqrt{\frac{\sum_{i=1}^{n}{x_{i}}^{2}}{n}}$$

The Mean metric (see Equation (4)) has been used to: recognize sitting and standing [28,29]; it discriminates between periods of activity and rest [30]; and as an input to classifiers such as Decision Table, KNN, J48, Naïve Bayes, Random Forest, Hidden Markov Model (HMM) [7,16,31,32]:

(4) $${\text{Mean}}_{X-\text{Axis}}=\frac{\sum_{i=1}^{n}x_{i}}{n}$$

The next metric, SMA is used to distinguish between periods of activity and rest in order to identify when the subject is mobilizing and undertaking activities, and when they are immobile [15,33,34]. Equation (5) implements SMA:

(5) $$\text{SMA}=\frac{\sum_{i=1}^{n}(\left|x_{i}\right|+\left|y_{i}\right|)}{n}$$

SMV, normally referred to as Signal Vector Magnitude (SVM) but changed to SMV to avoid confusion with the SVM classifier used, indicates the degree of movement intensity and is an essential metric in fall detection [33,34]. The SMV value is calculated using Equation (6):

(6) $$\text{SMV}=\frac{\sum_{i=1}^{n}\sqrt{{x_{i}}^{2}+{y_{i}}^{2}}}{n}$$

Two additional metrics used in this research are Energy and Entropy, which discriminate between types of ADL such as walking, standing still, running, sitting and relaxing [7,35]. The calculation of the Energy value is based on Equation (7) and the Entropy is calculated using the Matlab function from [36]:

(7) $${\text{Energy}}_{x-\text{Axis}}=\frac{\sum_{i=1}^{n}FFT_{x_{i}}^{2}}{n}-{\text{Mean}}_{X-\text{Axis}}$$

Another feature that was extracted from the accelerometer data stream is the FFTPeak for each axis. The metric has been used for activity recognition [5,12,35]. The FFTPeak algorithm was based on the Matlab Example found at [37].

The last metric used is Standard Deviation (STD), which has been extensively used for activity recognition [29]; and as an input to classifiers, such as J48, Random Forest and Artificial Neural Networks (ANN) [16,38]. Equation (8) describes the calculation:

(8) $${\text{STD}}_{X-\text{Axis}}=\sqrt{\frac{\sum_{i=1}^{n}{\left(x_{i}-{\text{Mean}}_{X-\text{Axis}}\right)}^{2}}{n}}$$

### 3.4. Classifier Selection

The software tool Weka implements the algorithms of several different classifiers from which nine were selected based on literature to verify the effects of changes in the described parameters above. [12] points out that common algorithms for activity classification are Support Vector Machines (SVM), Decision Trees, and Bayesian classifiers. The work of [13] included the use of Decision Tress and Naïve Bayes classifier. [10] based their research on Decision Trees, Bagging of 10 Decision Trees, AdaBoost using Decision Trees as base classifiers and a Random Forest of 10 Decision Trees. Additionally, in the work of [16] the authors compare J48 (Decision Trees) and Random Forest. This investigation therefore included the following classifiers: Naïve Bayes, SMO (based on SVM), KNN, KStar, MultiClassClassifier, Bagging, Decision Table, J48, and Random Forest. All algorithms were tested using Weka's standard configuration and a 10-fold cross validation. An additional classifier fine-tuning is a research field in its own and therefore not discussed here. The use of different classification methods has enabled the authors to verify the impact of the sampling rate, segmentation method, and window size on the classification accuracy over a wide field of algorithms used in AAL.

## 4. Experimental Results

This section will be split in three subsections to focus on different aspects of the parameter selection problem introduced by the variation in classification methods (CM), sampling frequency (SF), segmentation methods (SM) and window size (WS). The first two analyses use an ANalysis Of VAriance (ANOVA) to investigate the impact of the identified parameters on the two dependent variables classification accuracy (CA, as described in Section 4.1) and computational load (CL, as described in Section 4.2). The analyses have been conducted in SPSS [39]. In the third subsection the authors present the results of an investigation into the effects of various input parameter combinations on CA and CL with a view to enable optimum parameter selection based on the identification of the corresponding Pareto curve (see Section 4.3).

**Figure 4.**
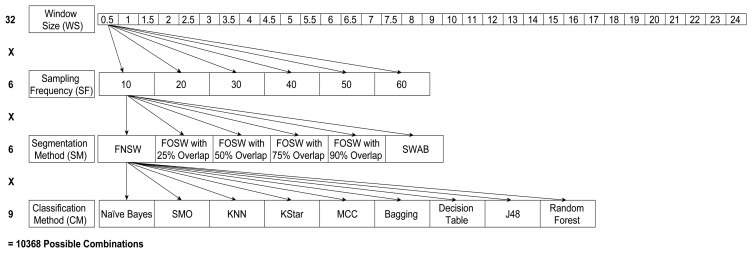
Parameter combinations for each classifier.

Figure 4 below shows the different levels of the four parameters CM, SF, SM, and WS; this has resulted in 32 different window sizes, three segmentation methods with different parameters (resulting in six SM levels) and six sampling frequencies for each of the nine different classification algorithms. This results in 10,368 different parameter levels for each of the 23 test subjects for the analysis of variance (ANOVA) in SPSS. The performance measures used are classification accuracy (CA) and computational load (CL), as explained in Section 4.2. Next to classification accuracy, literature also highlighted the use of precision, recall, and f-measure. In [40] the author argues that precision, recall, and f-measure are especially useful for highly imbalanced datasets. For example, when faced with a two-class problem with a split of 98% (majority) and 2% (minority), just guessing the majority class will achieve an accuracy of 98%. If the detection of the minority class, say, representing rare and infrequent events (e.g., falls), is important, an accuracy of 98% would be misleading in terms of the performance of the classifier. The datasets used for this research include six and four, respectively, different activity classes, which are roughly equally distributed and are equally important to classify. It is therefore adequate to use the accuracy of the classifier instead of f-measure:

(9) $$\text{Accuracy}=\frac{\text{Correct Classified Instances}}{\text{Total Instances}}*100$$

### 4.1. Statistical Analysis of Accuracy

This section reports on the impact of the variations of four input parameters (CM, SF, SM, and WS) on classification accuracy (output). During the initial analysis of the ANOVA output, the tested accuracy for the KStar algorithm showed a strong sensitivity to changes in the input parameters (SF, SM, and WS). Its influence on the analysis was significant and superimposed on the results. As a consequence the impact of certain input parameters appeared to be of significance for the classification accuracy, while overall the impact resulted from the sensitivity of the KStar classifier. Therefore the authors decided to exclude KStar in the analysis in order to avoid a misinterpretation of the overall impact of parameters on accuracy.

#### 4.1.1. Dataset Bao *et al.*

The ANOVA results (presented in Table 2), excluding KStar, showed that 49% of the variations in the dependent variable (accuracy) are described by the four input parameters. This means, that other input parameters that were not tested in the scope of this experiment may have further influence on the accuracy. Such a result is not surprising, as the investigated problem is highly complex and it is understandable that factors such as the test subject itself and the recorded movement have also an impact on the resulting accuracy.

The table shows the Sums of Squares (a measure for the average variability in the data), Degree of Freedom (df—scores that are free to vary once the mean of the set of scores is known), Mean Square (which is used to estimated the variance), F (F-Ratio represents the indicator for the significance on performance caused by the independent variables instead of chance), and Sig. (indicating the significance level at which the main/two-way interaction effects are significant <0.05 or non-significant >0.05) for all main and two-way interaction effects. The two-way interaction effects outline a changing main effect of one factor for different levels of a second factor and are therefore of higher interest than the main effect alone, if they are identified as being significant. The Sig. column in Table 2 shows that each main and the six two-way interaction effects (SM and CM, WS and CM, SM and WS, SF and WS, SF and CM, SF and SM) have a significant impact on the accuracy. Furthermore, the Type III Sum of Squares can be used as an indication for the importance of the main and two-way interaction effects. For an easier overview the rows are already sorted and it is observed that CM is the most influential factor followed by SM, WS, and SF in decreasing order. The significance for the two-way interaction effects starts with SM and CM and is followed in decreasing order by WS and CM, SM and WS, SF and WS, SF and CM and SF and SM.

**Figure 5.**
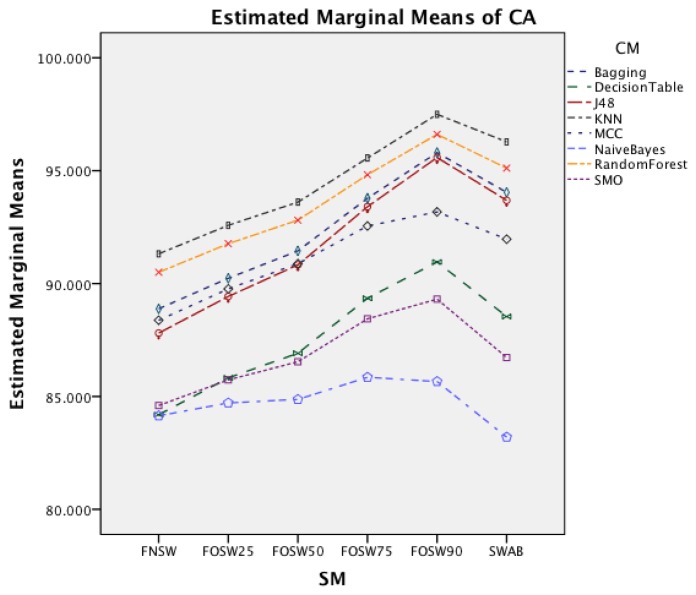
Two-way interaction effect for SM and CM.

Figure 5 shows the interaction effect of changes in the segmentation methods on the classifier methods. The Naïve Bayes classifier shows only a minor improvement in accuracy, whilst the remaining seven classifiers show a substantial increase with an increased segmentation overlap for FOSW. Another visible effect is the good performance of classifiers with the SWAB segmentation method, mostly outperforming FOSW with 75% overlap. For Naïve Bayes, SWAB showed a significant decrease in performance, which results in an even lower CA when compared to FNSW.

**Figure 6.**
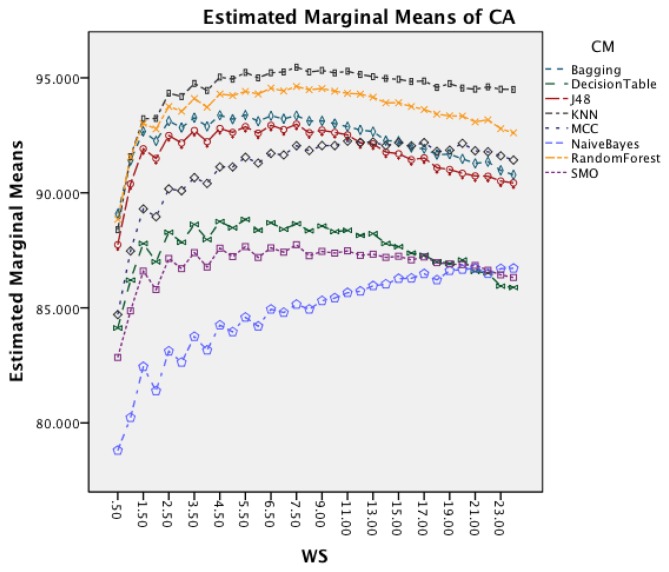
Two-way interaction effect for WS and CM.

Figure 6 presents the effect of an increased window size on the different classifiers. Besides Naïve Bayes, all classifiers show a decrease in accuracy for window sizes below 7 s before stagnating and start to decrease after the window size increases above 9 s. Naïve Bayes is the only CM that actually improves CA with an increased window size.

**Figure 7.**
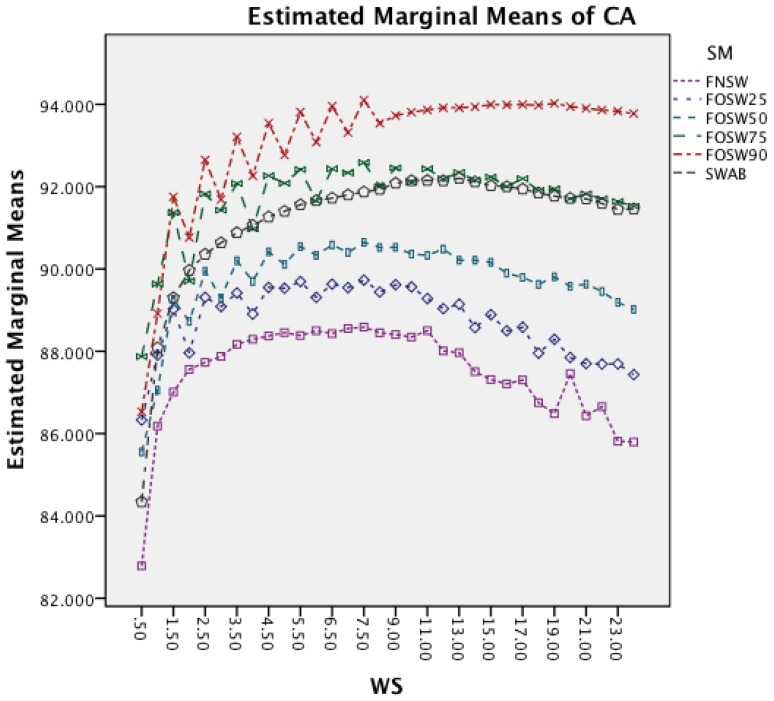
Two-way interaction effect for WS and SM.

The next effect investigated is the interaction between WS and SM in Figure 7. It is noticeable that an increased window size decreases the accuracy for each segmentation method. Furthermore, the effect reduces with an increased segmentation overlap, showing a less significant impact on FOSW with 90% compared to FOSW with 25% overlap. SWAB follows the behavior of FOSW with 75% overlap with a decreased overall accuracy. The figure also shows that a window size of 6.5 to 11 s results in the best accuracy.

**Figure 8.**
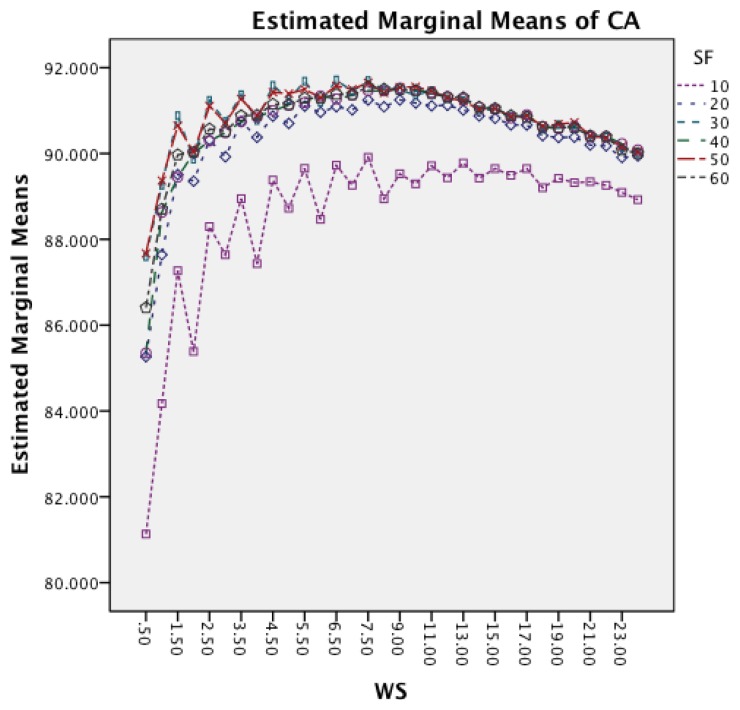
Two-way interaction effect for WS and SF.

Figure 8 shows the interaction effect between WS and SF. The effect of an increased window size is similar for all six sampling frequencies, while the effect is reduced for a sampling frequency of 10 Hz for longer window sizes. The graph also highlights that higher frequencies achieve the best accuracy for shorter window sizes, while the 10 Hz sampling frequency requires a slightly larger window.

The last effect under investigation is SF and CM. The graph is not presented, as there is no interaction effect between the different classifiers. The only effect that exists is a minor improvement of accuracy for a change of sampling frequency from 10 to 20 Hz with a nearly constant accuracy thereafter for all classifiers. This correlates with the statement in [13] that a sampling frequency above 20 Hz has only a marginally effect on the classification accuracy. For sampling frequencies above 20 Hz only minor improvements can be reported.

#### 4.1.2. Dataset Opportunity

The ANOVA results (presented in Table 3), excludes KStar for the same reason that is mentioned in Section 4.1.1, showed that 53% of the variations in the dependent variable (CA) are described by the four input parameters. As before, other input parameters that were not tested in the scope of this experiment may have further influence. Compared to the table earlier, the Sig. column shows this time, that the three two-way interaction effects (SF and SM, SF and CM, SF and WS) are non-significant for this dataset. The data is also sorted based on the Type III Sum of Squares for an easier overview of the importance of the main and two-way interaction effects. The influential factors of CM, SM, WS, and SF in decreasing order are the same compared to the earlier Table 2 but the order of the two-way interaction effects changed. The significant effect is now SM and WS, followed in decreasing order by SM and CM, and WS and CM with the rest non-significant.

**Figure 9.**
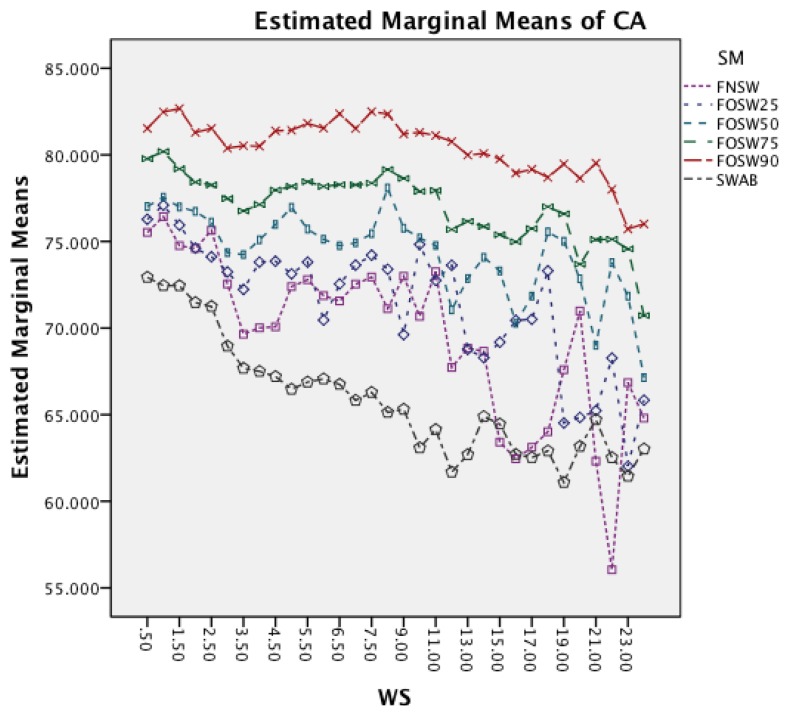
Two-way interaction effect for WS and SM.

Figure 9 investigates the interaction between SM and WS. It is noticeable that an increased window size decreases the accuracy for each segmentation method. While SWAB shows a near linear decrease in CA, the FOSW and FNSW segmentation methods, show higher variation in CA for longer WS.

**Figure 10.**
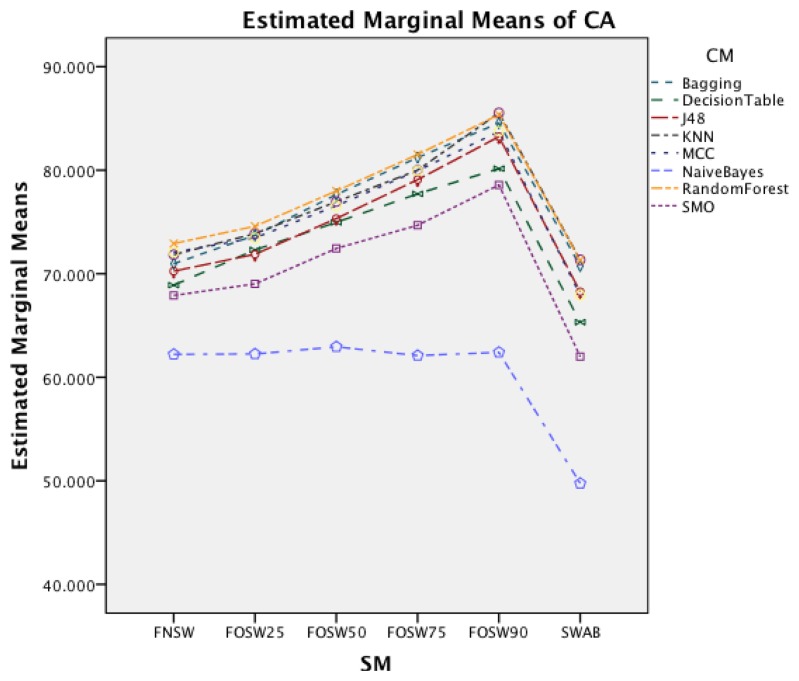
Two-way interaction effect for SM and CM.

Figure 10 shows the interaction effect of changes in the SM on the CM. The Naïve Bayes classifier shows only a minor improvement in accuracy, whilst the remaining seven classifiers show a substantial increase with an increased segmentation overlap for FOSW. Another visible effect is the poor performance of classifiers with the SWAB segmentation method for this dataset. Results are below FNSW, while earlier it was mostly outperforming FOSW with 75% overlap.

**Figure 11.**
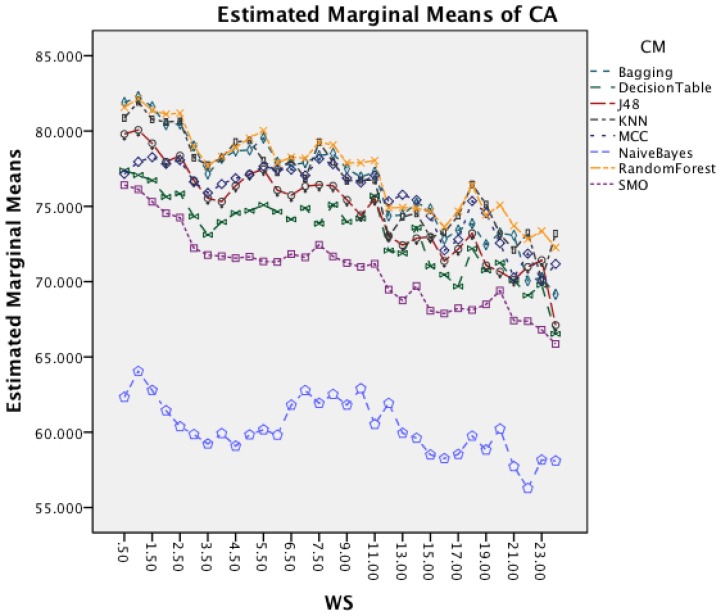
Two-way interaction effect for WS and CM.

The next effect investigated is the interaction between WS and CM in Figure 11. All CM show a decrease in CA for longer WS. The effect is less significant on Naïve Bayes compared to the other classifiers. The graph shows that shorter WS result in better CA result.

### 4.2. Statistical Analysis of Computational Load

**Figure 12.**
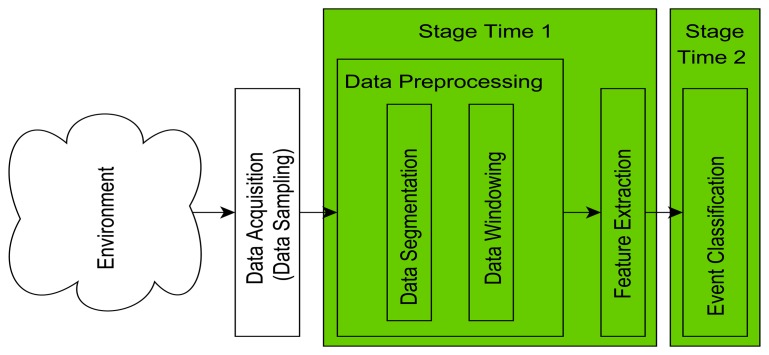
Timing factor for computational load.

The selection of different SF, SM, WS and CM does not only have an impact on the classification accuracy but also on the CL of the system. The CL for the classification of ADL events is based on two main factors. The first one is the data pre-processing and feature extraction step (indicated as *Stage_Time_*_1_ in Figure 12) and the second factor is the actual event classification (indicated as *Stage_Time_*_2_ in Figure 12).

*Stage_Time_*_1_ depends on the selected SF, SM and WS, excluding any other pre-processing steps such as filtering which is not of interest in this study, while *Stage_Time_*_2_ purely depends on the selected CM. For real-time applications, the combination of SM and WS introduces a limitation for certain parameter combinations, leading to the requirement that Equation (10) needs to be fulfilled:

(10) $${\text{Stage}}_{\text{Time}1}+{\text{Stage}}_{\text{Time}2}\leq \text{Max CL}$$

The authors therefore conducted an analysis with the CL as the dependent variable to investigate the influence of the four input parameter SF, SM, WS and CM. In the preliminary analysis, one of the levels of the SM input showed to have a high influence on the dependent variable. As before with KStar superimposing on parameters for the accuracy, the SWAB segmentation method increases noticeably the CL as compared to the other methods. Hence, effects that were non-significant before are significant once SWAB is removed as a SM level. Therefore, the analysis will outline the overall input variables without SWAB segmentation method.

#### 4.2.1. Dataset Bao *et al.*

The result of the ANOVA is represented in Table 4, outlining that 48% of the variation in the dependent variable CL are described by the variation in the input parameters. The Source column is sorted based on the Sum of Squares to allow for an easier recognition of the importance of an input parameter. The data highlights that the most important factor for the CL is CM (with SWAB included, this effect was actually non-significant). This is followed by WS, SF and as the least significant parameter SM. For the two-way interaction effect the five significant combinations are WS and CM, SM and CM, SF and WS, SM and WS followed by SF and SM.

**Figure 13.**
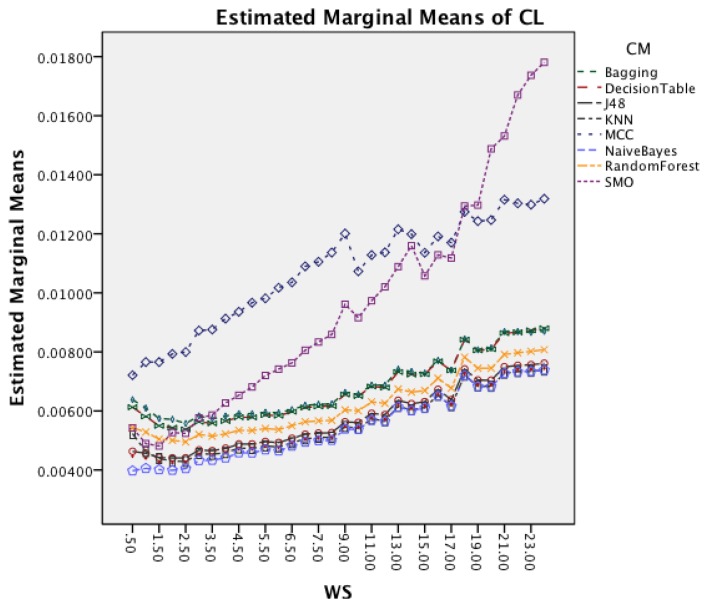
Two-way interaction effect for WS and CM.

Figure 13 shows the interaction effect between WS and CM. All classifiers require a longer CL for longer WS with a small visible drop in CL for short window sizes about 2 s. The graph also shows that the rate in which the CL increases is higher for MCC and SMO.

**Figure 14.**
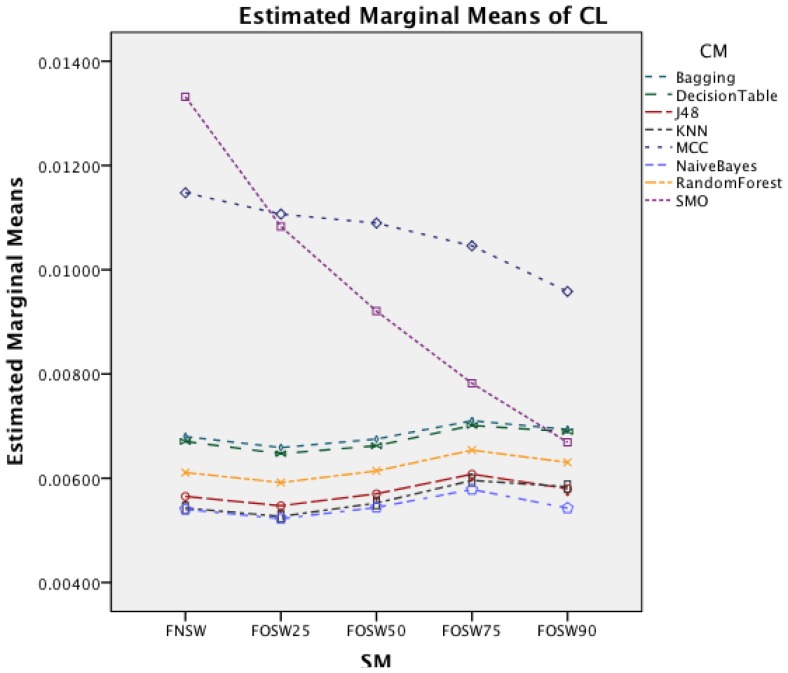
Two-way interaction effect for SM and CM.

The interaction effect for segmentation method and classifier method in Figure 14 shows that there is significant improvement in CL for MCC and SMO for an increased overlap. The remaining classifiers show only minor changes.

**Figure 15.**
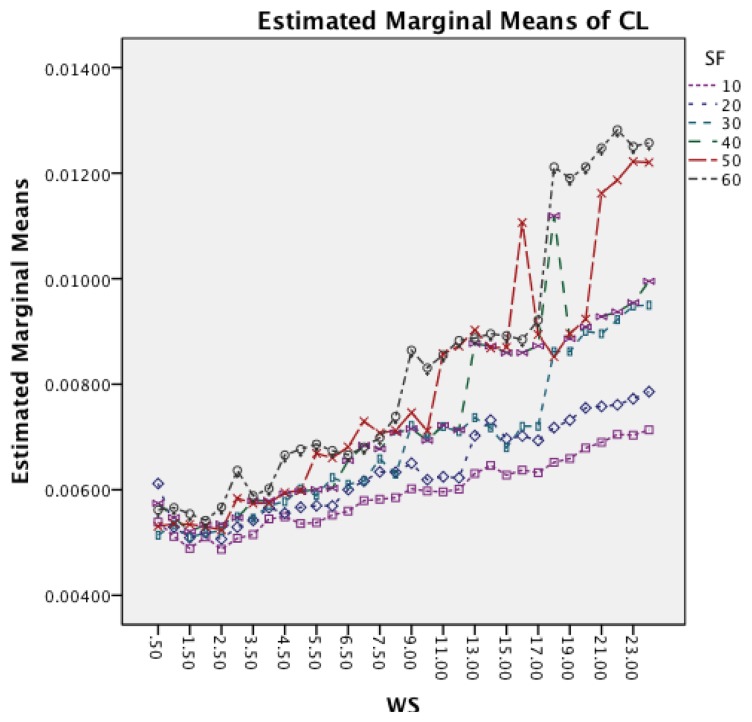
Two-way interaction effect for WS and SF.

Figure 15 shows the interaction effects between WS and SF. The longer window sizes result in a higher CL in all SF. Moreover, the graph shows that for higher SF the rate of increase in CL does also increase.

**Figure 16.**
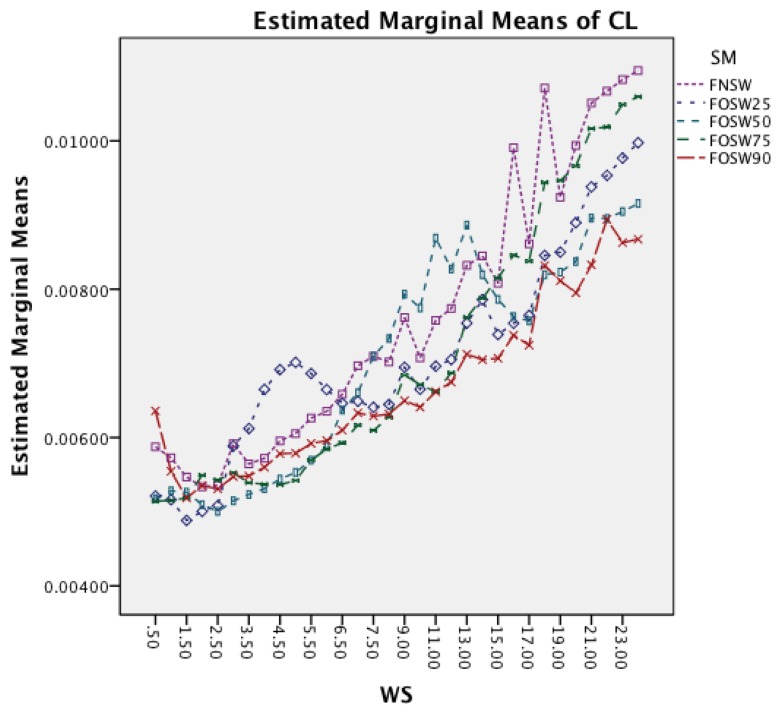
Two-way interaction effect for WS and SM.

The last interaction effect under investigation is WS and SM. The graph in Figure 16 shows that the segmentation method has similar patterns to the classifier. All segmentation methods have a significant increase in CL for longer window sizes. An interesting observation is that segmentation methods with higher overlap result in lower CL for higher window sizes.

#### 4.2.2. Dataset Opportunity

The result of the ANOVA is represented in Table 5, outlining that 72% of the variation in the dependent variable CL are described by the variation in the input parameters.

The Source column is sorted based on the Sum of Squares to allow for an easier recognition of the importance of an input parameter. The data highlights that the most important factor for the CL is CM. This is followed by WS, SF and as the least significant parameter SM. For the two-way interaction effect the four significant combinations are WS and CM, SM and CM, SF and WS, followed by SM and WS. The interaction effect SF and SM that was significant earlier, is non-significant for this dataset.

**Figure 17.**
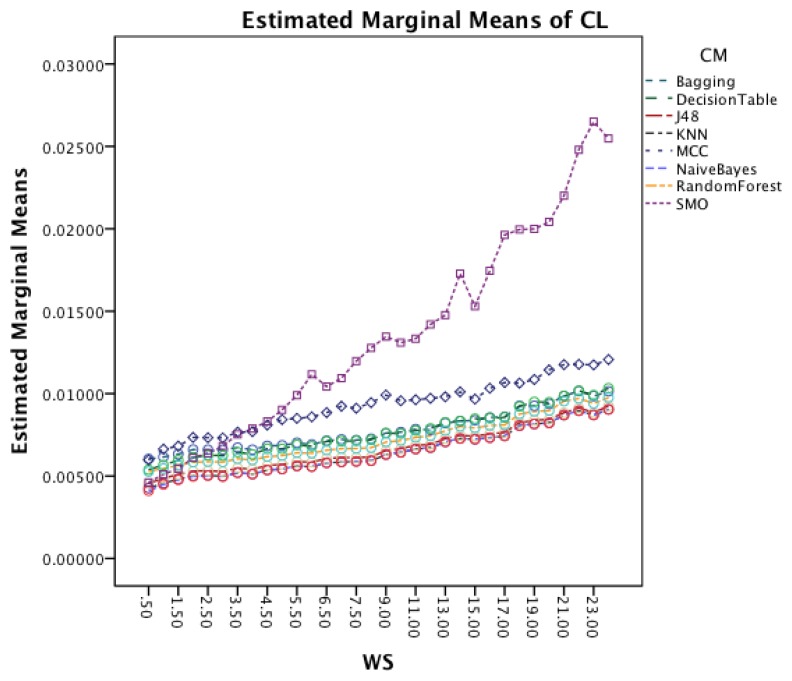
Two-way interaction effect for WS and CM.

Figure 17 shows the interaction effect between window size and classifiers. All classifiers require a longer CL for longer WS. The graph also shows that the rate in which the CL increases is higher for SMO. MCC that had an increased rate in the earlier dataset follows now the behavior of the other classifiers.

**Figure 18.**
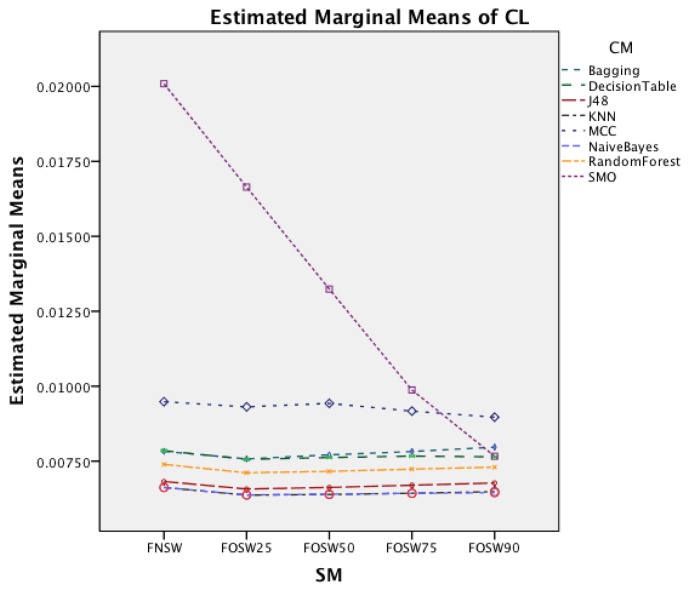
Two-way interaction effect for SM and CM.

The interaction effect for segmentation method and classifier in Figure 18 shows that there is significant improvement in CL for SMO for an increased overlap. The remaining classifiers show only minor changes for the different segmentation methods.

**Figure 19.**
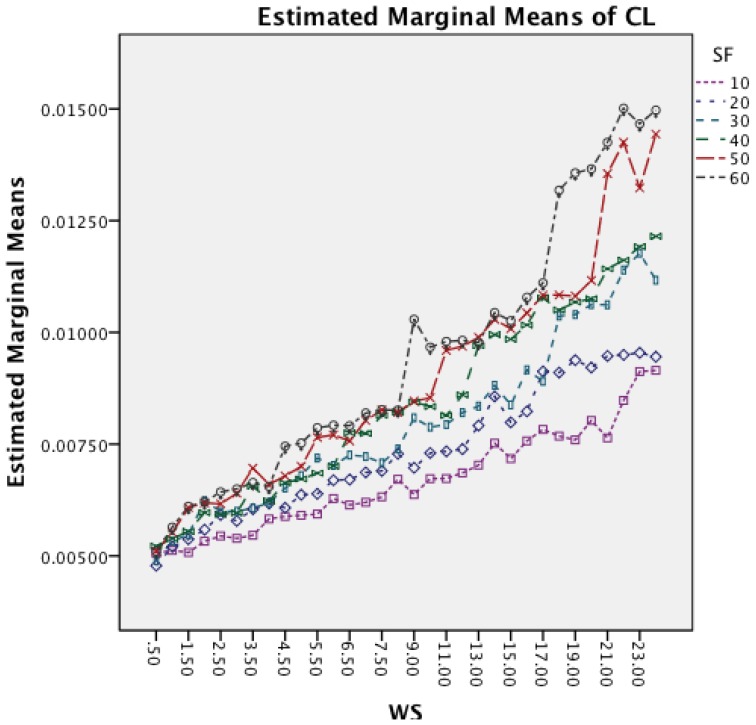
Two-way interaction effect for WS and SF.

Figure 19 shows the interaction effects between WS and SF. The longer window sizes result in a higher CL for all SF. Moreover, the graph shows that higher sampling frequencies result in an increased rate of CL as well.

**Figure 20.**
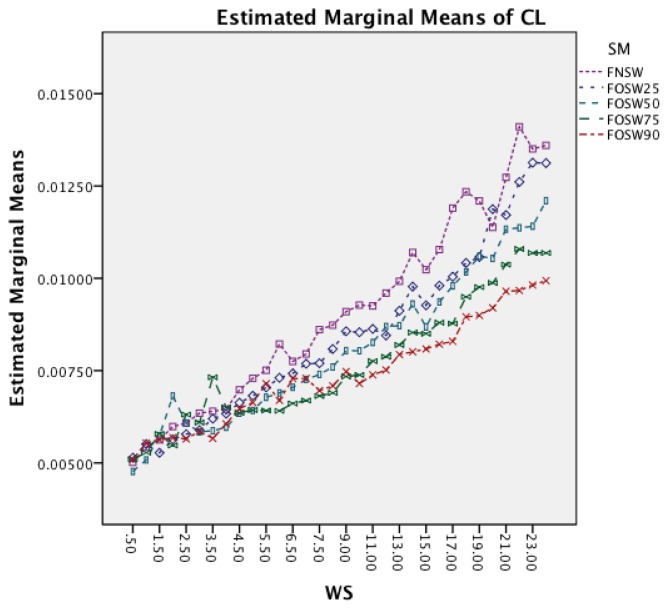
Two-way interaction effect for WS and SM.

The last interaction effect under investigation is windows size and segmentation method. The graph in Figure 20 shows that the segmentation method has similar patterns to the classifier. All segmentation methods have a significant increase in CL for longer window sizes. An interesting observation, as with the other dataset, is that the segmentation methods with higher overlap result in lower CL for higher window sizes.

### 4.3. Parameter Selection

**Figure 21.**
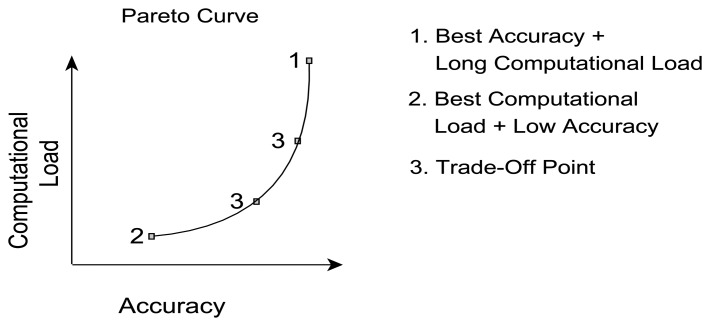
Explanation of the Pareto curve.

Based on the parameter influence described in Sections 4.1 and 4.2, the inevitable question still stands: what is the best parameter selection for a given requirement? The answer, however, depends strongly on the preference with respect to classification performance, e.g., is the best accuracy required or are there limitations to CL. Therefore, a set of well-performing parameter sets based on the trade-off between accuracy and CL were identified. For the given dataset certain parameter combinations will achieve a similar CA but will require different CL and vice versa. When plotted in a graph, such as presented in Figure 21, the best accuracy for a given CL would follow the black line (called Pareto frontier), with dominated parameter sets lying on the left hand side of the curve. Hence, a parameter set is dominated if there exists a combination of parameter values that results in the same level of accuracy with less CL or achieve better accuracy with the same CL. The Pareto frontier, also referred to as Pareto curve, outlines the set of non-dominated solutions, herein represented by a set of parameter combinations. One set of parameter values may achieve best CA at the cost of a high CL (Point 1) and another combination will achieve the lowest CL at the cost of a lower accuracy (Point 2). Parameter sets in between points 1 and 2 on the Pareto frontier are subject to a trade-off (Point 3), hence accepting the sacrifice of either, accuracy or CL, depending on the context of the applications or potential corresponding limitations, e.g., hardware constraints.

#### 4.3.1. Dataset Bao *et al.*

**Figure 22.**
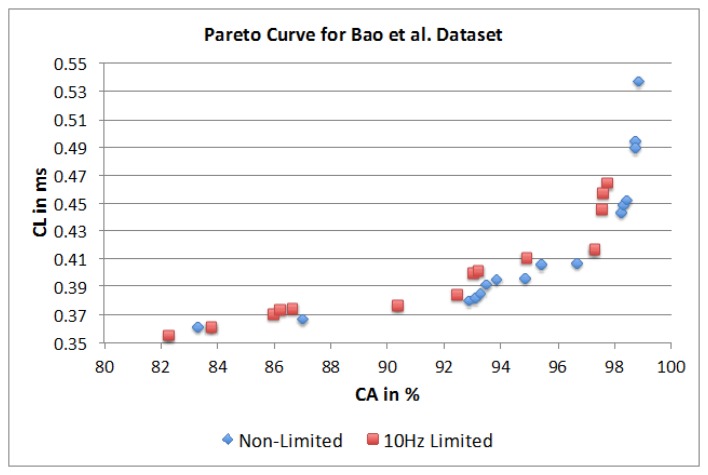
Dominant points on the Pareto curve.

Figure 22 represents two separate Pareto curves for the Bao *et al.* dataset. The illustration shows a non-limited Pareto curve over all 10,368 possible parameter combinations and a Pareto curve that is limited to 10 Hz sampling frequency, as this is a common hardware limitation for researchers using off-the-shelf components. For the non-limited Pareto curve, with 10,368 possible combinations, only 20 are dominating. The parameter combinations show a maximum SF of 30 Hz with equal occurrence of 20 and 30 Hz (8 combinations each). For the SM parameter only the FOSW method with various overlaps percentages shows to be of importance. An overlap value of 90% shows higher CA, while an overlap value of 25% results in shorter CL. The dominating points highlight short window sizes of 1.5 s for the shortest CL and a WS of 7.5 s for best CA. The dominating classifiers are Naïve Bayes and KNN. The latter is in the majority of combinations and results in the best CA, while the former results in the shortest CL. The achievable CA ranges from 82.2% with a CL of 0.355 ms to 98.8% with a CL of 0.537 ms. The parameter combinations for the Pareto curve limited to 10 Hz SF, highlights only 14 dominating parameter combinations. The SM shows similar behavior to the non-limited dataset, as well as a higher influence of the FOSW method with 75% overlap. The WS is around 1.5 s for shortest CL and around 7.5 s for best CA. The CM parameter for the 10 Hz limited case is nearly identical to the non-limited case; with the exception of the presence of J48 as an influential algorithm in the former case. The achievable CA ranges from 82.2% with a CL of 0.355 ms to 97.7% with a CL of 0.464 ms.

#### 4.3.2. Opportunity Dataset

**Figure 23.**
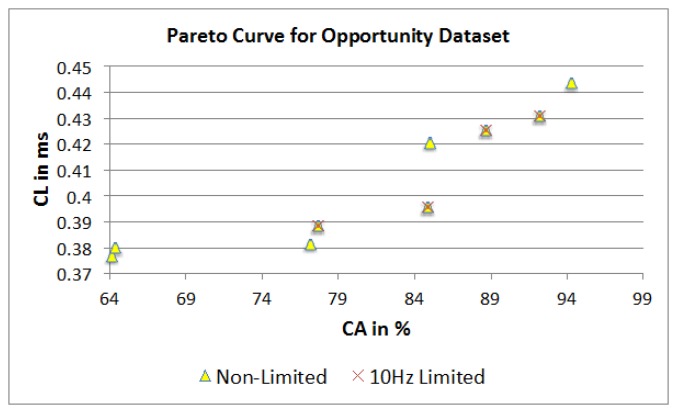
Dominant points on the Pareto curve.

Figure 23 represents the two separate Pareto curves for the Opportunity dataset, based on a non-limited and 10 Hz limited parameter combination set. For the non-limited Pareto curve, 12 dominating points are identified, while the 10 Hz limited dataset shows only six dominating points. The full dataset shows SF parameter values in the range of 10 to 30 Hz with a majority split between 10 and 20 Hz. FOSW with 90% overlap (SM) dominates for higher CA and with 25% overlap for lower CL in both datasets.

Short WS have a significant influence in this dataset, as both datasets include parameter combinations with 1.5 s or less. The dominating classifiers are Naïve Bayes, and KNN. This is a slight variation compared to the 10 Hz limited Bao *et al.* dataset. The non-limited dataset has a range of 64.2% to 94.3% in CA with a CL of 0.376 to 0.443 ms. The 10 Hz limited dataset ranges from 63.2% to 92.2% for CA with 0.383 to 0.43 ms for CL.

## 5. Summary and Discussion of Results

One of the main problems in AAL is the availability (or the lack thereof) of test subjects, as compared to clinical trials, where subjects can reach into the thousands. In [41] the authors highlight that research in AAL starts out as a demonstration of feasibility under laboratory conditions, which in a further step needs an increased number of participants and ethical considerations. In [42], the authors argue that the use of any one of two activity classification methods, uniform (where the training data comes from all tests subjects) and individual (training data representing separate test subjects) can lead to problems; generalization (arising from the uniform method), small training data set (individual) can both result in poor performance. The research and associated experiments presented here fall in the individual category as performance measures (CA and CL) are generated for each of the test subjects involved. The authors believe that despite the pitfalls described above, this was the better method to adopt; this is in line with Elbert *et al.*' approach. Moreover, as the tested activities are nearly equally represented in the dataset, using the accuracy measure can be done without loss of validity. This is in contrast with the differentiation between, say, normal and abnormal conditions, where the latter occurs rarely resulting in an imbalanced set of data; the use precision, recall and f-measure would be a more appropriate performance indicator [20].

In summary the outputs of the work presented here, are listed below:

- The importance of parameters for CA ranked in order of decreasing influence is CM, SM, WS and SF;
- The impact of WS is different for both datasets;
- Increased segmentation overlap improves CA;
- The influence of SWAB on CA is different in both datasets;
- SF above 10 Hz has only a minor improvement on CA;
- CL behaves the same for both dataset;
- The importance of parameters for CL ranked in order of decreasing influence is CM, WS, SF and SM;
- Some dominant parameter combinations of the Pareto curve are similar for both datasets;
- Higher CL does not automatically result in higher CA.

The following discussion will look into the results of the ANalysis Of VAriance (ANOVA) for CA and CL and finish with the dominant parameter points of the Pareto curves. The two-way interaction effect between SM and CM highlights for both datasets that FOSW with 90% overlap results in the best CA. From FNSW (no overlap) to FOSW with 90% overlap, both datasets show that more overlap improves the CA. A possible reason for this is that the increase in overlap allows for a bigger training set and has the lowest loss of information, in the range of investigated SM. The results for SWAB are mixed. For the Bao *et al.* dataset CA is just below CA for FOSW with 90% overlap, while the Opportunity dataset showed SWAB to be the worst segmentation method tested. Further research needs to look into the actual benefit of a dynamically sliding window, which incidentally was reported in [20] as giving good results, as the results reported here (in terms of classification accuracy) are inconsistent between the two datasets. Another difference between the datasets is observed for the WS and CM two-way interaction effect. While for the first dataset (Bao *et al.*), the CA improves for window sizes between 1 and 8 s and only decrease for WS values above 8 s, the second dataset (Opportunity) achieves best CA for 0.5 s and starts to decrease immediately after that. A similar behavior can be seen for the two-way interaction effect of WS and CM and WS and SM with both datasets (compare Figure 6 and Figure 7 in Section 4.1.1 and Figure 11 and Figure 9 in Section 4.1.2). Researchers should therefore choose smaller window sizes if possible. Another difference between the two datasets is the significance of the two-way interaction effect WS and SF; namely, significant interaction (but not the most significant) for Bao *et al.* and non-significant interaction for the Opportunity dataset. The graph in Figure 8 (see Section 4.1.1) shows that sampling frequencies above 10 Hz achieve nearly the same CA, while the 10 Hz sampling frequency is marginally lower, endorsing the finding in [13] that sampling frequencies above 20 Hz result in only minor accuracy gains.

For CL, both datasets show the same behavior for the two-way interaction effects. For the three interaction effects including WS (WS and CM, WS and SM, WS and SF) similar behavior is observable. A shorter WS results in a lower CL, while a longer WS will increase the CL. This effect is lowest for WS and CM and highest for WS and SM. The interaction effect between SM and CM highlights no significant change for any classifier besides SMO. SMO is the only classifier that can reduce the CL with an increased segmentation overlap.

**Figure 24.**
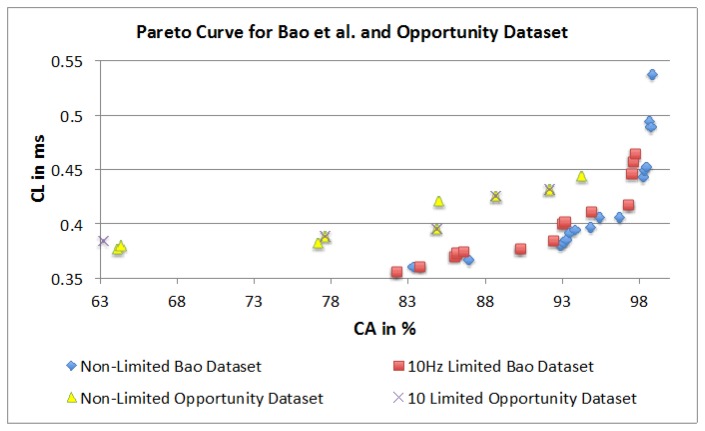
Dominant points on the Pareto curve for both datasets.

The authors used ANOVA to quantify the influence of the different parameters on the CA and CL. They have also used a Pareto curve based approach to highlight dominant parameter combinations for “optimum” achievable performance (optimality being decided by the user in a given context/application). Figure 24 presents the four Pareto curves based on the dominant combinations. The illustration shows that all graphs have a similar outline and that it is possible to achieve similar results irrespective of the dataset. This is highlighted with only a 4.3% difference in CA between the two top performing parameter combinations. However, the dominant parameter combinations are different for each dataset. Therefore, it is not possible to present a single combination that will work best for all datasets. Having said that, some dominant points have similar parameter combinations. In both datasets high CA is achieved with the KNN classifier and the FOSW with 90% overlap for SM. Furthermore, the Pareto points show that a sampling frequency above 30 Hz is not necessary and only minor improvements in CA are achieved with a sampling frequency above 10 Hz. As a consequence, the authors recommend adjusting parameters individually for each dataset and test subject to achieve optimal results, especially with regards to WS. The Pareto curves also reveal that a higher computational load does not necessarily result in better classification accuracy, as the algorithms under investigation are not recursive. The Pareto curve is also a good tool to investigate the influence of a hardware limitation such as a low sampling rate, storage space and battery runtime. When superimposing the hardware limited Pareto curve with the non-limited curve a simple comparison of achievable CA and CL is possible. The results presented in Section 4.3 in combination with the ANOVA in Sections 4.1 and 4.2 can be used for future research as a tool to select parameter combinations for AAL event classifications with the sound understanding of how each parameter influences the outcome of event classification accuracy and computational load.

## 6. Conclusions and Future Work

This paper has presented a new instrument to help select data capture and processing parameters for the recognition of Activities of Daily Living (ADL). A review of the literature uncovered a lack of consensus in terms of the selection of sampling frequency, segmentation method and window size, and classifier method for the recognition of ADL. The impact of the sampling frequency (six levels), segmentation method (three segmentation algorithms with different parameters resulting in six different levels) and segmentation window size (32 levels) on the classification accuracy and computational load of a set of commonly used classifiers (nine levels) has been investigated. This has involved experimenting with two datasets, containing 20 and three test subjects, respectively, and analysis of the resulting data using ANalysis Of VAriance (ANOVA). The analysis showed that the choice of classifier method is the most important parameter followed by the segmentation method, window size and finally sampling frequency. It also showed that in the case of computational load the parameters ranked in order of decreasing influence are classifier method, window size, sampling frequency, and segmentation method. The results have been presented graphically using a Pareto curve, which highlighted two dominant classifiers for both datasets (KNN, Naïve Bayes). The Pareto curve did not show matching dominant points in both datasets, however, it showed that combinations of three out of the four factors (CM, SM, SF) are likely to result in dominant points. The authors have suggested that the Pareto curve is a good instrument which can be used to select sets of parameters based on their impact on classification accuracy and computational load and resolve trade-off issues.

As part of their future work in the general area of AAL, the authors plan to investigate a number of issues specific to the findings presented in this paper. An important point of interest is the identification of the reasons behind the inconsistency between the two datasets used in terms of the influence of WS on the classification accuracy. A possible influential factor, not considered in the present work, is the nature of the ADL itself. It might be necessary to adjust the WS parameter with regards to the expected ADLs in the dataset; [16] suggested to use different WS parameter combinations per activity. The authors also intend to investigate the influence of the features extracted and the position of sensors on classification accuracy. Different feature combinations (and a reduction in the number of required features) may improve the classification accuracy of different ADLs as well as reduce the CL. Moreover, the authors' propose to couple the results obtained so far with a Decision Support System (DSS). Having the option to learn, adjust from past experiences, and include new ADLs, would allow for more informed decisions in parameter selection over time. Additionally, hardware limitations, such as battery time and communication bandwidth, should be included into the selection process. Another direction that the authors want to pursue is the investigation of how to improve the Pareto curve by replacing the computational load with a measure for training time and training samples, as it could highlight classifiers that could achieve good accuracy within a low starting time.

## Figures and Tables

**Table 1.** Inconsistency in sampling rates and segmentation windows for AAL.

**Authors**	**Sampling Frequency [Hz]**	**Segmentation Window [s]**	**Segmentation Method**	**Testsubject Information**	**ADLs**
Huynh [5]	512	0.25, 0.5, 1, 2, 4	FNSW, FOSW 50%, FOSW 75%, FOSW 80.5%, FOSW 93.75%		Walking, Standing, Jogging, Skipping, Hopping, Riding Bus
Sekine [6]	256			Subjects 11; Age 69.3 ± 5.6 years; Height 1.54 ± 0.078 m; Weight 50.4 ± 9.6 kg	Walking
Bao [7]	76.25	6.7	FOSW 50%	Subjects: 13 male, 7 female; Age 17–48 years	Walking, Sitting & Relaxing, Standing Stil, Watching TV, Running, Stretching, Scrubbing, Folding Laundry, Brushing teeth, Riding Elevator, Walking Carrying items, Working on Computer, Eating or Driniking, Reading, Bicycling, Strength Training, Vacuuming, Lying Down & Relaxing, Climbing Stairs, Riding Escalator
Preece [8]	64	2 and 3	FOSW 50%	Subjects: 10 male, 10 female; Age 31 ± 7 years; Height 1.71 ± 0.07 m; Weight 68 ± 10 kg; BMI 24 ± 3	Walking, Walking Upstairs, Walking Downstairs, Hopping on Left Leg, Hopping on Right Leg, Jumping
Wang [9]	50	2.56	FOSW 50%	Subjects: 39 male, 12 female; Age 21–64 years; Height 1.53–188 m; Weight 42–94 kg	Walking, Walking Slope Up, Walking Slope Down, Walking Stairs Up, Walking Stairs Down
Casale [10]	52	1	FOSW 50%	Subjects: 11 male, 3 female	Walking Stairs Up, Walking Stairs Down, Walking, Talking, Staying Standing, Working at Computer
Ravi [11]	50	5.12	FOSW 50%	Subjects 2	Standing, Walking, Running, Walking Stairs Up, Walking Stairs Down, Situps, Vacuuming, Brushing Teeth
Pärkkä [12]	50	5		Subjects 7; median (range); 27 years (4–37); Height 180 (92–187)	Lying, Sitting, Standing, Walking, Bicycling, Running
Maurer [13]	50	4	FOSW 92%	Subjects 6	Sitting, Standing, Walking, Walking Stairs Up, Walking Stairs Down, Running
Antonsson [14]	1–30			Subjects 12	Walking (Gait)
Bouten [15]	20			Subjects: 13 male; Age 27 ± 4 years; Height 1.83 ± 0.07 m; Weight 77 ± 12 kg	Sedentary Activities, Household Activities, Walking
Gjoreski [16]	5	1.4			Standing, Lying, Sitting, On all fours, Sitting on the Ground, Going Down, Standing Up
Nyan [21]	256	2		Subjects 22; Age 20–45 years; Height 1.67–1.94 m; Weight 45–93 kg	Walking, Walking Upstairs, Walking Downstairs
Kasteren [22]		60	FNSW	Subject 1	Leaving House, Toileting, Showering, Sleeping, Breakfast, Dinner, Drink
Patterson [23]		74		Subject 1	Using Bathroom, Making Oatmeal, Making Soft-Boiled Eggs, Preparing Orange-Juice, Making Coffee, Making Tea, Making or Answering a Phone Call, Taking out the Trash, Setting the Table, Eating Breakfast, Clearing Table
Pietka [3]			FNSW, FOSW, SAX, SM		
Keogh [17]			FNSW, FOSW, Bup, SWAB		
Chu [18]			RbW		
Kozina [19]			Dwin		
Ortiz Laguna [20]			VSW		

**Table 2.** ANOVA output for the CA as the dependent variable (Tests of Between-Subjects Effects. Dependent Variable: CA).

**Source**	**Type III Sum of Squares**	**df**	**Mean Square**	**F**	**Sig.**
Corrected Model	3,257,844 ^a^	670	4,862	260	0.000
Intercept	1,503,713,645	1	1,503,713,645	80,275,936	0.000
SF	116,380	5	23,276	1,243	0.000
WS	216,554	31	6,986	373	0.000
SM	650,201	5	130,040	6,942	0.000
CM	1,961,904	7	280,272	14,962	0.000
SF * SM	5,924	25	237	13	0.000
SF * CM	7,591	35	217	12	0.000
SF * WS	36,024	155	232	12	0.000
SM * WS	60,930	155	393	21	0.000
WS * CM	92,164	217	425	23	0.000
SM * CM	110,091	35	3,145	168	0.000
Error	3,439,779	183,633	19		
Total	1,510,410,864	184,304			
Corrected Total	6,697,622	184,303			

a R Squared = 0.486 (Adjusted R Squared = 0.485).

**Table 3.** ANOVA output for the CA as the dependent variable (Tests of Between-Subjects Effects. Dependent Variable: CA).

**Source**	**Type III Sum of Squares**	**df**	**Mean Square**	**F**	**Sig.**
Corrected Model	1,785,658 ^a^	670	2,665	45	0.000
Intercept	147,805,613	1	147,805,613	2,587,861	0.000
SF	9,501	5	1,900	33	0.000
WS	183,628	31	5,923	104	0.000
SM	639,572	5	127,914	2,240	0.000
CM	759,536	7	108,505	1,900	0.000
SF * SM	778	25	31	0.545	0.968
SF * CM	1,445	35	41	0.723	0.886
SF * WS	4,898	155	32	0.553	1.000
WS * CM	25,038	217	115	2	0.000
SM * CM	70,397	35	2,011	35	0.000
SM * WS	90,865	155	586	10	0.000
Error	1,540,791	26,977	57		
Total	151,132,062	27,648			
Corrected Total	3,326,449	27,647			

a R Squared = 0.537 (Adjusted R Squared = 0.525).

**Table 4.** ANOVA output for the CL as the dependent variable (Tests of Between-Subjects Effects. Dependent Variable: CL).

**Source**	**Type III Sum of Squares**	**df**	**Mean Square**	**F**	**Sig.**
Corrected Model	1.316 ^a^	626	0.002	222	0.000
Intercept	7.755	1	7.755	818,571	0.000
SM	0.013	4	0.003	355	0.000
SF	0.104	5	0.021	2,202	0.000
WS	0.330	31	0.011	1,122	0.000
CM	0.515	7	0.074	7,769	0.000
SF * CM	0.000	35	3.587 × 10^−6^	0.379	1.000
SF * SM	0.001	20	5.456 × 10^−5^	5.759	0.000
SM * WS	0.034	124	0.000	29	0.000
SF * WS	0.082	155	0.001	56	0.000
SM * CM	0.103	28	0.004	390	0.000
WS * CM	0.134	217	0.001	65	0.000
Error	1.449	152,957	9.474 × 10^−6^		
Total	10.521	153,584			
Corrected Total	2.766	153,583			

a R Squared = 0.476 (Adjusted R Squared = 0.474).

**Table 5.** ANOVA output for the CL as the dependent variable (Tests of Between-Subjects Effects. Dependent Variable: CL).

**Source**	**Type III Sum of Squares**	**df**	**Mean Square**	**F**	**Sig.**
Corrected Model	0.358 ^a^	626	0.001	93	0.000
Intercept	1.525	1	1.525	247,087	0.000
SM	0.008	4	0.002	328	0.000
SF	0.021	5	0.004	671	0.000
WS	0.090	31	0.003	470	0.000
CM	0.112	7	0.016	2,595	0.000
SF * SM	0.000	20	7.884 × 10^−6^	1	0.181
SF * CM	0.000	35	7.236 × 10^−6^	1	0.223
SM * WS	0.006	124	4.866 × 10^−5^	8	0.000
SF * WS	0.011	155	6.966 × 10^−5^	11	0.000
SM * CM	0.050	28	0.002	290	0.000
WS * CM	0.060	217	0.000	45	0.000
Error	0.138	22,413	6.172 × 10^−6^		
Total	2.021	23,040			
Corrected Total	0.496	23,039			

a R Squared = 0.721 (Adjusted R Squared = 0.714).
